# Reproductive factors, intima media thickness and carotid plaques in a cross-sectional study of postmenopausal women enrolled in the population-based KORA F4 study

**DOI:** 10.1186/1472-6874-14-17

**Published:** 2014-01-24

**Authors:** Doris Stöckl, Annette Peters, Barbara Thorand, Margit Heier, Wolfgang Koenig, Jochen Seissler, Joachim Thiery, Wolfgang Rathmann, Christa Meisinger

**Affiliations:** 1 Institute of Epidemiology II, Helmholtz Zentrum München, German Research Center for Environmental Health, Neuherberg, Germany; 2 Department of Obstetrics and Gynaecology, Campus Grosshadern, Ludwig-Maximilians-University, Munich, Germany; 3 Central Hospital of Augsburg, MONICA/KORA Myocardial Infarction Registry, Augsburg, Germany; 4 Department of Internal Medicine II – Cardiology, University of Ulm Medical Center, Ulm, Germany; 5 Medizinische Klinik und Poliklinik IV, Diabetes Zentrum - Campus Innenstadt, Klinikum der Ludwig-Maximilians-Universität, München and Clinical Cooperation Group Diabetes, Ludwig-Maximilians-Universität München and Helmholtz Zentrum München, Munich, Germany; 6 Institute of Laboratory Medicine, Clinical Chemistry and Molecular Diagnostics, University of Leipzig, Leipzig, Germany; 7 Institute of Biometrics and Epidemiology, German Diabetes Center, Leibniz Center for Diabetes Research at Heinrich Heine University, Düsseldorf, Germany

**Keywords:** Intima media thickness, Atherosclerosis, Cardiovascular disease, Women, Reproductive factors, Gender studies

## Abstract

**Background:**

Reproductive events may affect the onset of chronic diseases. We examined the possible association between reproductive parameters and intima media thickness (IMT) or carotid plaques in the common carotid artery in a population-based sample.

**Methods:**

This cross-sectional study analysed data of 800 postmenopausal women aged 50 to 81 years of the population-based KORA F4 study, conducted between 2006 and 2008 in Southern Germany. Reproductive parameters were obtained by standardised interviews.

**Results:**

Age at menarche below 12 years compared to 12-15 years was significantly associated with carotid plaques (age-adjusted OR 2.23, 95% CI 1.13-4.43, p-value 0.018, multivariable adjusted 2.11, 1.05-4.26, 0.037), but not with IMT. Ever use of hormone replacement therapy was inversely associated with carotid plaques (age-adjusted 0.60, 0.44-0.81, p = 0.001, multivariable-adjusted 0.62, 0.45-0.86, 0.003) and IMT in the age-adjusted model (mean 0.89, 95% CI 0.88-0.90, p = 0.033) but not in the multivariable-adjusted model (mean 0.89, 95% CI 0.88-0.90, p = 0.075). Parity, age at menopause, time since menopause, duration of fertile period, current use of hormone replacement therapy, ever use of oral contraceptives, hysterectomy, bilateral oophorectomy, hot flashes and depressive mood in relation to the menopausal transition were not associated with carotid plaques or IMT.

**Conclusion:**

Our study showed, that there may be an independent association between the reproductive parameters age at menarche and ever use of hormone replacement therapy with carotid plaques in the common carotid artery, but not with IMT. Further research, especially in studies with prospective population-based study design, is necessary to assess in detail what events in women’s life lead to increased IMT or CP.

## Background

The identification of risk factors or risk markers for prevention of cardiovascular disease (CVD) represents an important research area. Subclinical atherosclerosis, a risk factor for cardiovascular events, can be assessed non-invasively by ultrasonography of the carotid arteries by assessing intima media thickness (IMT) and the presence of plaques (CP). The presence and progression of IMT and CP are associated with the development of coronary artery disease events to some extend [1-4]. Yet, a recently published meta-analysis found CP to be more accurate than IMT in predicting these events [5].

CVD affects women nearly as often as men. In earlier adulthood, women are more protected, but after menopause the sex difference diminishes [6], suggesting an effect of female hormones with possible protection of the premenopausal hormonal status. So far, there is a lack of gender specific studies on CVD risk, treatment and prognosis.

Reproductive events during the life-span of women have been shown to be associated with chronic diseases. Lower age at menarche is associated with diabetes [7], CVD [8], metabolic syndrome [9] and mortality [8]. Parity was shown to be associated with CVD [10] and the metabolic syndrome [11,12]. The menopausal transition is associated with CVD [13] and hormone replacement therapy (HRT) in form of estrogen and progesterone application increases the risk of CVD [14]. But HRT has been reported to have an initially protective effect on the calcification of the coronary arteries [15].

There are only a few prior studies on whether reproductive factors are associated with IMT or CP, which are markers for early atherosclerosis and appear before the onset of symptomatic CVD. A population-based study of 746 Finish women aged 45-74 showed that of all examined reproductive factors, only stillbirth was associated with an increased risk of atherosclerotic plaques [16]. This study has examined various reproductive factors, but for example age at menarche was not examined. Other studies have only examined selected reproductive factors and their association with IMT and so far there are only few population-based studies. Oophorectomy before natural menopause [17] and hot flashes, as a sign of the vasomotor symptoms during menopausal transition have been found to be associated with an increased IMT [18,19], but other studies don’t support this association [20]. Parity has been related to increased IMT [21] in younger [22] as well as in elderly women [23].

The aim of this study is to investigate whether there is an association between a variety of reproductive parameters and IMT and CP in the common carotid artery (CCA) in postmenopausal women from the general population.

## Methods

### Subjects

The KORA F4 (2006-2008) study is the 7-year follow-up study of the KORA S4 (1999-2001) study, a population-based health survey performed in the city of Augsburg and their two surrounding counties in Southern Germany. The abbreviation KORA stands for Cooperative Health Research in the Region of Augsburg. The target population for the S4 baseline study consists of all German residents of the region aged 25 to 74 years. Of the randomly selected 6640 subjects, 4261 (64.2%) participated in the S4 baseline study. The study design, sampling method and data collection have been described in detail elsewhere [24].

Of the 4261 participants in S4, 3080 took part in the F4 follow-up study. Participants were not invited for F4 if they were deceased (n = 176, 4%), relocated outside the study region, were lost to follow-up (n = 206, 5%) or had demanded deletion of their address data (n = 12, 0.2%). Of the remaining 3867 eligible persons, the contact failed for 174, 218 were not able to participate in the study because they were too ill or had no time, and 395 denied participation in the follow-up. The final response rate was 79.6%.

For the current analysis all postmenopausal women aged 50 to 81 years at follow-up (n = 940) were included.

From this sample, we excluded 140 females for whom no or incomplete information on CP, carotid IMT or any of the covariables was available. Therefore, this analysis finally included 800 study participants. These 140 excluded women did not differ significantly regarding age, BMI and other main charactersistcs from the included 800 women.

The investigations were carried out in accordance with the Declaration of Helsinki, including written informed consent of all study participants. The study was approved by the Ethics Committee of the Bavarian Medical Association.

### Data collection

Information on sociodemographic variables, smoking habits, physical activity level, medication use, reproductive parameters and alcohol consumption were gathered by trained medical staff during a standardized interview. Education was estimated by recording years of schooling completed. In addition, all study participants underwent a standardized medical examination. All measurement procedures have been described in detail elsewhere [24]. Anthropometric measurements were performed after subjects had removed their shoes, heavy clothing and belts. Body height was measured to the nearest 0.1 cm and weight to the nearest 0.1 kg. BMI was calculated as weight [kg] divided by height^2^ [m^2^]. Waist circumference (WC) was measured at the level midway between the lower rib margin and the iliac crest. Actual hypertension was defined as blood pressure values greater than 140/90 mmHg or the use of antihypertensive medication, given that the subjects were aware of being hypertensive. Individuals who participated in leisure time physical activity during summer and winter and were active for at least one hour per week in either season were classified as being physically active. Women who consumed more than 20 g alcohol per day were regarded as heavy alcohol drinkers. A fasting venous blood sample was obtained from all study participants while sitting. The parameters total cholesterol, triglycerides and fasting blood glucose were used for the analysis. Fasting blood glucose was analyzed using a hexokinase method (GLU Flex of Dade Behring, Germany), fasting triglycerides (TG) with the TGL Flex GPO-PAP assay of Dade Behring and the analysis of total cholesterol wase carried out using the analyzer Dimension RxL (Dade Behring, Germany).

### Assessment of reproductive parameters

Reproductive parameters were obtained through personal interviews by trained medical staff in the KORA S4 and F4 surveys and have been described previously [25]. Most variables were obtained in both interviews and underwent rigorous quality assessment for internal validity. For this analysis mainly data of the KORA F4 study were used.

Women were classified postmenopausal at the absence of menstrual bleeding for 12 consecutive months, if they had bilateral oophorectomy (either alone or in combination with hysterectomy) and had hysterectomy without bilateral oophorectomy and were above 50 years (without reported menopause before hysterectomy). This was asked in the S4 and F4 interview. Age at menarche was defined as age at the first menstrual bleeding, assessed in full years. The question, which was asked only in the baseline S4 study was open-ended: “At what age did you have your first menstrual period (menarche)?” Women were asked in S4 and F4 to recall their number of pregnancies and live-born children, if they ever used oral contraceptives or HRT. Parity was defined as the number of reported deliveries. At the examination date in the F4 study, all subjects were asked to bring their current medication to the study center and the barcode of each medication was scanned. From these data the category current use of HRT was constructed. There was no further differentiation in the type of HRT, e.g. with or without progestin use. Ever use of oral contraception or HRT was assessed in the interviews. Furthermore the women were asked about the presence of two symptoms of the menopausal transition: hot flashes and depressive mood. Age at menopause was inquired in both surveys and is only available for 602 women. The high amount of missing values is due to the difficult assessment of this variable, because ongoing vaginal bleeding due to HRT and hysterectomy early in life without bilateral oophorectomy while still being premenopausal, make an age determination challenging and some women simply don’t remember their age at menopause. From the variable age at menopause the variables time since menopause (current age minus age at menopause) in years and fertility duration (age at menopause minus age at menarche) in years are constructed.

### Vascular examination

Ultrasound measurement of IMT was performed as described previously [26]. Briefly, CCAs were scanned in angles of 60°-180° (right CCA) and 210°-300° (left CCA) using a high resolution ultrasound system (Sonoline G50, Siemens Medical Solutions, Munich, Germany or Acuson X300, Siemens Medical Solutions, Munich, Germany). Optimal images of the far wall were recorded on DVD videotapes. Measurements of IMT were performed off-line over a length of 10 mm beginning at 0-5 mm of the dilatation of the distal CCA using an automated edge detection reading system (Prowin software, Medical Technologies International, USA) by one certified reader. The mean of the left and right CCA were used to calculate IMT.

Plaques were defined as a distinct area with either rising into the lumen or mineralization. CP assessment was performed from the DVD videotapes which were recorded for the IMT measurement. Therefore only plaques in the CCA were assessed. No further vessels were scanned for plaques.

### Statistical analyses

Basic characteristics of the study population were analyzed for the whole sample and stratified for carotid plaques. For normally distributed variables the mean and standard deviation and for categorical variables percentages were calculated. Two models were fitted, the first one controlling for age, the second model uses a multivariable approach with age, BMI, education, family status, physical activity, smoking, alcohol consumption, fasting blood glucose levels, total cholesterol and triglycerides as potential confounder. For the selection of confounder a literature research was performed, which showed that age, BMI, social status (education and family status were considered in our study to represent social status), physical activity, alcohol consumption and smoking were included as confounder in comparable studies [1,16,27]. Generalized linear models were used for the analysis of IMT and logistic regression analyses for CP.

Significance tests were two-tailed and p-values less than 0.05 were considered statistically significant. All analyses were performed using SAS (version 9.3, SAS Institute Inc, Cary, NC, USA).

## Results

This study comprises 800 postmenopausal women aged 50 to 81 years. Basic characteristics of the whole study sample and according to the presence or absence of CP in the CCA are presented in Table 1. Women with CP were older and more likely to suffer from hypertension or diabetes. They were less likely to be heavy alcohol drinkers and were less physically active. Women without CP were more likely to have a history of oral contraceptive or HRT use.

**Table 1:** Selected characteristics of the study population for the whole sample and stratified for carotid plaques in the common carotid artery

	**Whole study population**	**No plaque**	**Plaque**	**p-values**
n	800	498	302	
**Age** (years)	64.2 (8.2)	62.0 (7.6)	67.9 (8.0)	<0.0001
**IMT** (mm)	0.895 (0.124)	0.856 (0.109)	0.961 (0.119)	<0.0001
**Waist circumference** (cm)	91.4 (12.5)	90.7 (12.9)	92.5 (11.8)	0.051
**BMI** (kg/m^2^)	28.4 (5.1)	28.2 (5.2)	28.7 (4.9)	0.143
**BMI at age 25 years** (kg/m^2^)	22.5 (3.3)	22.4 (3.4)	22.7 (3.2)	0.300
**Family status** (%)				0.075
Single	5.3	4.8	6.5	
Married	66.8	69.4	61.6	
Divorced or widowed	28.0	25.8	32.0	
**Education** < 10 years (%)	62.3	57.2	70.1	0.0003
**Systolic BP** (mmHg)	121.6 (18.1)	120.6 (16.6)	125.0 (19.8)	0.001
**Diastolic BP** (mmHg)	73.6 (9.2)	74.5 (8.8)	72.5 (9.7)	0.003
**History of diabetes** (%)^1^	13.2	11.2	16.7	0.029
**Actual hypertension** (%)^2^	44.8	40.7	55.8	<0.0001
**Current use of antihypertensive medication** (%)	41.1	33.5	53.7	<0.0001
**Total cholesterol** (mg/dl)	230.6 (38.5)	62.6 (14.6)	61.0 (14.8)	0.951
**Fasting glucose** (mg/dl)	98.7 (17.3)	97.5 (15.3)	100.8 (20.0)	0.010
**Triglycerides** (mg/dl)	121.3 (73.9)	119.0 (77.4)	125.2 (66.8)	0.251
**Alcohol > 20 g/day** (%)	16.0	16.1	15.0	0.079
**Current smoking** (%)	10.9	11.2	10.9	0.844
**Physically active** (%)	56.0	60.1	48.0	0.001
**Menarche** (years)	13.6 (1.7)	13.6 (1.6)	13.8 (1.8)	0.104
**Parity** (%)				0.195
0	11.8	10.3	14.6	
1-2	48.1	49.8	44.9	
>2	40.2	39.9	40.5	
**Age at menopause** (years)^3^	49.0 (5.0)	48.7 (5.1)	49.3 (4.8)	0.143
**Time since menopause** (years)^3^	15.8 (9.0)	13.8 (8.6)	18.9 (8.7)	<0.0001
**Duration of Fertility** (years)^3^	35.3 (5.1)	35.1 (5.2)	35.5 (5.0)	0.322
**Current HRT** (%)	14.4	16.9	10.5	0.010
**Ever HRT** (%)	59.0	64.0	50.3	<0.0001
**Ever OC** (%)	61.6	66.7	54.8	0.0005
**Hysterectomy** (%)	33.8	34.7	32.0	0.286
**Bilateral oophorectomy** (%)	6.8	7.0	6.5	0.687
**Hot flashes** (%)	43.2	48.3	34.7	<0.0001
**Depressive mood** (%)	37.1	35.7	37.8	0.695

Data presented as mean (standard deviation) or percentage respectively.

*Abbreviations*: BMI: Body Mass Index, BP: Blood pressure, HRT: Hormone replacement therapy, OC: oral contraceptives.

p-values are presented for the difference of present and absent plaques.

^1^ missing information for 13 women.

^2^ actual hypertension was defined as current use of blood pressure lowering medication or a blood pressure ≥140/90 mmHg, given that the subjects were aware of being hypertensive.

^3^ n = 602, due to missing values of age at menopause.

The mean IMT of all women included in this analysis was 0.895 mm with a standard deviation of 0.124. Table 2 presents the associations between the examined reproductive parameters and IMT. Ever use of HRT, but not current use, was associated with IMT in the age-adjusted model (mean 0.89, 95% CI 0.88-0.90, p-value 0.033). After further adjustment for additional risk factors statistical significance was lost. There was no significant interaction between current use of HT and time since menopause (p-value 0.711). Parity, age at menopause, time since menopause, duration of fertile period, current use of hormone replacement therapy, ever use of oral contraceptives, hysterectomy, bilateral oophorectomy, hot flashes and depressive mood in relation to the menopausal transition were not associated with IMT.

**Table 2:** Intima media thickness (mm) and reproductive parameters in 800 postmenopausal women from the KORA F4 study

	**Age-adjusted**			**Multivariable-adjusted**		
	**Mean**	**95% CI**	**p-value**	**Mean**	**95% CI**	**p-value**
**Menarche**			0.701			0.873
<12 years	0.906	0.879-0.933		0.902	0.875-0.929	
12-15 years	0.895	0.886-0.903		0.894	0.886-0.903	
>15 years	0.892	0.872-0.914		0.896	0.875-0.917	
**Parity**			0.114			0.407
0	0.876	0.854-0.898		0.883	0.860-0.905	
1-2	0.895	0.884-0.906		0.895	0.884-0.906	
>2	0.902	0.890-0.914		0.900	0.888-0.912	
**Age at menopause**^**1**^			0.766			0.590
≥ 50 years	0.903	0.890-0.915		0.904	0.891-0.916	
< 50 years	0.900	0.887-0.913		0.899	0.886-0.912	
**Time since menopause**^**1**^			0.136			0.079
> 15 years	0.891	0.875-0.907		0.889	0.873-0.905	
≤ 15 years	0.911	0.895-0.928		0.913	0.897-0.929	
**Duration of fertility**^**1**^			0.645			0.683
> 35.0	0.903	0.891-0.913		0.903	0.891-0.915	
≤ 35.0	0.899	0.886-0.909		0.899	0.886-0.912	
**Current use of HRT**			0.817			0.711
Yes	0.893	0.873-0.913		0.899	0.879-0.919	
No	0.896	0.887-0.904		0.895	0.887-0.903	
**Ever use of HRT**			0.033			0.075
Yes	0.888	0.878-0.898		0.890	0.880-0.900	
No	0.905	0.893-0.917		0.902	0.891-0.914	
**Ever use of OC**			0.767			0.394
Yes	0.896	0.886-0.906		0.898	0.888-0.908	
No	0.894	0.880-0.907		0.891	0.878-0.904	
**Hysterectomy**			0.370			0.277
Yes	0.890	0.877-0.903		0.888	0.875-0.901	
No	0.898	0.888-0.907		0.899	0.890-0.908	
**Bilateral oophorectomy**			0.775			0.614
Yes	0.891	0.862-0.920		0.888	0.859-0.917	
No	0.896	0.888-0.903		0.896	0.888-0.904	
**Hot flashes**			0.684			0.452
Yes	0.893	0.881-0.905		0.892	0.880-0.904	
No	0.897	0.886-0.907		0.898	0.888-0.908	
**Depressive mood**			0.753			0.989
Yes	0.897	0.884-0.909		0.895	0.883-0.906	
No	0.894	0.885-0.904		0.895	0.886-0.905	

*Abbreviations*: HRT: Hormone replacement therapy, OC: oral contraceptives.

Generalized linear models.

Estimated means of IMT are presented.

Multivariable-adjusted: Age, BMI, education, family status, physical activity, smoking, alcohol consumption, total cholesterol, triglycerides and fasting blood glucose.

^1^n = 602, due to missing values of age at menopause.

Table 3 displays the association between the reproductive parameters and CP in the CCA, assessed by logistic regression analysis. Ever use of HRT, but not current use, was significantly inversely associated with CP (age-adjusted: OR 0.60, 95% CI 0.44-0.81, and multivariable-adjusted: OR 0.62, 95% CI 0.45-0.86) and lower age at menarche was associated with CP (below 12 years, compared to 12-15years) (age-adjusted: OR 2.23, 95% CI 1.13-4.43, multivariable-adjusted: OR 2.11, 95% CI 1.05-4.26). All other examined reproductive parameters showed no significant association with CP.

**Table 3:** Carotid plaques in the common carotid artery and reproductive parameters in 800 postmenopausal women, the KORA F4 study

	**Age-adjusted**			**Multivariable-adjusted**		
	**OR**	**95% CI**	**p-value**	**OR**	**95% CI**	**p-value**
**Menarche**						
<12 years	2.23	1.13-4.43	0.018	2.11	1.05-4.26	0.037
12-15 years**						
>15 years	1.22	0.77-1.92	0.290	1.25	0.79-1.99	0.436
**Parity**						
0	1.37	0.83-2.26	0.195	1.41	0.83-2.38	0.186
1-2	1.01	0.73-1.41	0.397	1.02	0.73-1.42	0.383
>2**						
**Age at menopause**^**1**^						
≥ 50 years	0.94	0.66-1.33	0.714	1.00	0.70-1.45	0.962
< 50 years**						
**Time since menopause**^**1**^						
> 15 years	0.97	0.58-1.63	0.921	0.91	0.54-1.53	0.772
≤ 15 years**						
**Duration of fertility**^**1**^						
> 35.0 years	0.84	0.59-1.20	0.339	0.87	0.61-1.25	0.449
≤ 35.0 years**						
**Current use of HRT**						
Yes	0.78	0.49-1.24	0.294	0.79	0.49-1.26	0.316
No**						
**Ever use of HRT**						
Yes	0.60	0.44-0.81	0.001	0.62	0.45-0.86	0.003
No**						
**Ever use of OC**						
Yes	1.25	0.89-1.78	0.203	1.34	0.93-1.90	0.125
No**						
**Hysterectomy**						
Yes	0.84	0.61-1.16	0.285	0.83	0.60-1.16	0.284
No**						
**Bilateral oophorectomy**						
Yes	0.68	0.37-1.26	0.221	0.65	0.35-1.22	0.178
No**						
**Hot flashes**						
Yes	0.93	0.67-1.30	0.664	0.90	0.64-1.26	0.535
No**						
**Depressed mood**						
Yes	1.16	0.84-1.60	0.367	1.15	0.83-1.59	0.396
No**						

Logistic regression analyses.

*Abbreviations*: HRT: Hormone replacement therapy, OC: oral contraceptives

**reference group.

Multivariable-adjusted: Age, BMI, education, family status, physical activity, smoking, alcohol consumption, total cholesterol, triglycerides and fasting blood glucose.

^1^n = 602, due to missing values of age at menopause.

## Discussion

The present cross-sectional study shows that only a few reproductive parameters are associated with IMT and CP in the CCA, both early markers of atherosclerosis. There was an age-adjusted association of ever use of HRT with decreased IMT and reduced presence of CP, however, statistical significance remained only for CP after multivariable adjustment. An age at menarche below 12 years was associated with increased presence of CP, but not IMT. All other examined reproductive factors did not show an association, neither with CP nor IMT.

The findings in this study support the results from other studies in which early age at menarche has been shown to be associated with premature CVD [8], cardiovascular risk factors like metabolic syndrome [9], diabetes [7] as well as peripheral arterial disease (PAD) [28]. The underlying reasons for these associations are not completely understood. Earlier onset of menarche could be due to obesity in childhood. It has been shown that obese children who stayed obese in adolescence and young adulthood had an increased risk of carotid-artery atherosclerosis, as well as hypertension, dyslipidemia and type 2 diabetes. This effect could not be shown in obese children who were normal weight in adulthood. Their risk was comparable to never obese individuals [29]. Thus, it might be assumed that obesity has an effect on the association between early age at menarche and early signs of atherosclerosis, such as IMT or CP. However, in the present study, earlier age at menarche was independently of BMI associated with the presence of CP. This finding needs to be confirmed or refuted in further large, particularly prospective population-based studies. Another pathway for the association of age at menarche with CP might be due to genetic traits. Recent genome-wide association studies showed obesity loci to be associated with age at menarche, suggesting complex genetic interrelationships [30].

In the present study ever use of HRT was associated with a lower risk of CP in the CCA, even after multivariable adjustment, while current use of HRT was not associated with CP. A cross-sectional study in 538 postmenopausal women in France, Belgium and the Netherlands showed that ever users of HRT had smaller and thinner carotid artery walls than nonusers with a lower rate of progression of subclinical atherosclerosis [31]. These findings are in partial contrast to findings from large observational studies like the Nurses’ Health study or the randomized double-blinded Women’s Health Initiative (WHI) [14,15]. Differences between observational studies and randomized trials have been argued to be due to the selection of women. It is known that women who chose to take HRT compared to women who did not, differ in certain characteristics like education, social status, and the prevalence of chronic diseases in observational epidemiologic studies [31]. The WHI showed that users of HRT have less calcification than nonusers, but they state that estrogen has complex biologic effects and may influence the risk for cardiovascular events and other outcomes through multiple pathways [15]. Recently published results show that there was no decreased risk of CHD in subgroups of women, e.g. who started HRT within 10 years after menopause. But subgroups were small [14]. We tried to adjust for all possible confounders, but residual confounding cannot be entirely excluded. Therefore our finding of an association between ever use of HRT and CP needs to be interpreted carefully. Hot flashes during the menopausal transition have been associated with increased IMT in some studies [18,19], but other studies did not see this association [20,32]. The reasons for these conflicting results may be due to different study designs (e.g. assessment of hot flashes, inclusion and exclusion criteria) and differences in the study sample (e.g. age, position in the menopausal transition, cardiovascular risk factors). The association of hot flashes with CP has to our knowledge not been described before.

Premenopausal hormonal influence is expected to be protective of CVD. In early adulthood women are more protected than men, but after menopause this sex difference diminishes. Reproductive factors which occur over the life span of women do influence plasma levels of female hormones. This effect is well observed during pregnancy. Compared to non-pregnant women there are marked differences in the hormone levels with metabolic and functional changes. Cardiovascular regulations have been shown to change early in pregnancy, persist postpartum and seem to be enhanced during subsequent pregnancy [33]. The authors speculated that the changes during pregnancy might lower cardiovascular risk in later life. The findings in this study, where no associations between parity, and duration of fertility and IMT were found, are in contrast to other studies [21-23]. Wolff et al showed in another German population-based study (SHIP study) that nulliparity and a higher number of children were associated with increased IMT [21]. They speculated that this association was partially mediated through conventional risk factors and socioeconomic determinants.

Genome-wide association studies have recently found no evidence that SNP´s associated with coronary artery disease are associated with carotid IMT and therefore suggested that genetic associations are not acting via early vessel remodeling or early atherosclerosis [34].

IMT is a less reliable marker for early atherosclerosis than CP. IMT measurements are widely used in clinical practice to assess early atherosclerosis due to its non-invasive technique. But IMT without presence of CP represents mainly hypertensive medial hypertrophy and has a lower sensitivity to predict cardiovascular events [5]. A recent meta-analysis has shown that CP, compared with carotid IMT, more accurately predicts coronary artery disease events, like myocardial infarction [5]. The study showed that the absence of CP provides greater assurance with 10-year myocardial infarction event rates of 4.0% (95% CI 3.6–4.7%) [5].

The advantage of our study is the population-based design. The study sample was drawn randomly from the general population. Another strength is the collection of the data by experienced examiners according to standardized protocols. The sonographers did not know about the cardiovascular history of the subjects and measurements underwent rigorous quality control. Multiple reproductive factors have been assessed. Furthermore this study uses mean IMT values, which are better reproducible and less susceptible to outliers compared to maximal carotid IMT measurements which reflect more advanced stages with focal thickening [5]. Limitations are the cross-sectional design; therefore cause and effect relationships cannot be stated. Screening for CP was only performed in the CCA during the measurement of IMT. This is a severe limitation for the results of our study, because no further vessels were screened for the prevalence of plaques, a procedure which has been recommended by Inaba et. al.: Carotid IMT assessment should always be supplemented by a thorough scan of the extra cranial carotid arteries for CP assessment to increase the diagnostic performance of carotid ultrasound [5]. Nevertheless we think that the results of our study are interesting and worth to be presented despite the lack of systematic plaque assessment because there are very few studies published who examined the association of reproductive parameters and CP. Another limitation of the study is that information on CVD events was not yet available for the analysis. Furthermore, some reproductive variables were categorized rather broad, like hot flashes. In the KORA F4 study the women were only asked if they currently suffer from hot flashes, without asking for the duration or severity. The same applies to current use or past use of HRT. The categories were yes/no without differentiation on application forms, dosages and formulas. A recall bias cannot be ruled out due to the assessment of reproductive variables in the interview.

## Conclusion

Our study showed a significantly positive association of early menarche with CP in the common carotid artery and a significant inverse association of ever use of hormone replacement therapy and CP. There was no independent association between any of the investigated reproductive parameters and IMT. The findings of this study are explorative and possibly due to chance and need to be proven in further population-based studies with a bigger sample size. Further research, especially in studies with prospective population-based study designs, is necessary to assess in detail what events in women’s life lead to increased IMT or CP.

## Abbreviations

BMI: Body mass index; BP: Blood pressure; CCA: Common carotid artery; CP: Carotid plaques; CVD: Cardiovascular disease; HRT: Hormone replacement therapy; IMT: Intima media thickness; OC: Oral contraceptives.

## Competing interest

The authors declare that they have no competing interest.

## Authors’ contribution

DS planned the study, performed the data analysis and wrote the manuscript. CM contributed to the acquisition of data, planning of the study, the performance of the data analysis and reviewed and edited the manuscript. BT, AP, JT, WK, JS, WR and MH contributed to the acquisition of data and reviewed and edited the manuscript. All authors have viewed and approved the final version of the manuscript.

## Pre-publication history

The pre-publication history for this paper can be accessed here:

http://www.biomedcentral.com/1472-6874/14/17/prepub
